# Clinical and Laboratory Diagnosis of Dermatophilosis (Cutaneous Streptothricosis) in Cattle in Ethiopia: Case Report

**DOI:** 10.1002/vms3.70245

**Published:** 2025-02-06

**Authors:** Dessalew Habte, Habtamu Addis, Kifle Wondimagegnehu

**Affiliations:** ^1^ Department of Veterinary Laboratory Technology Debre Markos University Debre Markos Ethiopia; ^2^ Department of Veterinary Laboratory Technology Injibara University Injibara Ethiopia

**Keywords:** bull, clinical diagnosis, dermatophilosis, *Dermatophilus congolensis*, skin

## Abstract

This study aims and documents the clinical and laboratory diagnosis of bovine dermatophilosis, caused by *Dermatophilus congolensis* that causes exudation and matting of hairs and wools with the formation of crusts and scab. An adult local breed bull with a history of reduced appetite, pruritus, dirty scabs and crusts as initial lesion coalesce on its different body parts was presented to Debre Markos multipurpose veterinary clinic. The rectal temperature and other vital parameters were within the normal range. The clinical signs observed were exudative dermatitis forming crusts and scabs, pruritus, matted hair like a paintbrush and keratinized material creating wart‐like lesions that were widely distributed in different body parts. Few ticks were observed on the skin of the bull suspected as vector of the disease. Culture of skin scraps on blood agar showed small, grayish‐white raised granular and hemolytic colonies. Staining of skin scraps by Giemsa and Gram's stain and cultured colony by Gram's stain indicated the characteristics of *Dermatophilus congolensis*, etiology of dermatophilosis. Other laboratory tests also showed the characteristics of *D. congolensis*. The case was treated by penstrep and ivermectin parentally and iodine tincture topically, and the bull showed progressive clinical improvement and complete recovery within 2 months. In conclusion, dermatophilosis is an economically important skin disease, highly prevalent in Ethiopia, and needs early detection and treatment by penstrep intramuscularly and ivermectine subcutaneously with iodine tincture topically in line with proper husbandry practices to control the losses.

## 1 Introduction

Dermatophilosis (Cutaneous actinomycosis) is a chronic tick‐borne bacterial skin disease affecting multiple species of animals which is characterized by crustiness and exudates accumulating at the base of the hair or wool fibers. It affects more severely cattle, sheep and goats with minor zoonosis to the public. This disease occurs throughout the tropical and temperate regions of the world most commonly in wet weather or rainy season climatic conditions (Walter et al. 2017). Different factors like dipping, shearing or introducing an infected animal into a herd or flock can increase the spread of infection. Besides its impact on leather quality, it impose economic losses as a result of reduction in body weight gain and milk yield, occasional mortality, reduction of performance in working animals and losses associated with treatment and prevention of the disease (Aliye 2020; Ndhlovu and Masika 2016).

The disease is caused by *Dermatophilus congolensis* in the family Dermatophilaceae bacteria which is characterized as Gram‐positive, spore‐forming, non‐acid‐fast, facultative anaerobic and branching actinomyces. *Dermatophilus congolensis* has distinct life cycle and exists in two characteristic morphologic forms as branched hyphae and motile zoospores. It requires damage to the skin and invade hair follicles, sweat glands and other epidermal structures for infection (Marsella 2014; Swanson, Petran, and Hanlin 2018). It can be transmitted through direct contact or by vectors (ticks, sheep kids and flies) and through contaminated instruments. Moisture or cold weather and the presence of ectoparasites in association with skin abrasion are predisposing factors to transmit the disease during rainy season (Covarrubias 2015).

Clinical signs include regional or generalized tufted papules initially, matted hair like paintbrush, pruritus, thick and horny crusts projecting from the skin that vary in color from cream to brown (2–5 cm in diameters) and keratinized material forming wart‐like lesions that can have a wide distribution mainly on the dorsum, face, neck, distal extremities, udder and scrotum (Tresamol et al. 2015). Dermatophilosis can be diagnosed by history, clinical signs, microscopic examinations of stained exudates or skin scrapings with Giemsa and/or Gram's stain, cytology, culture on blood agar media, Fluorescent Antibody Test, Enzyme Linked Immunosorbent Assay and Polymerase Chain Reaction. It can be differentially diagnosed in cattle from photosensitization, mange mite, warts, lumpy skin disease and dermatophytosis (Jackson et al. 2018; Olaogun and Jeremiah 2018).

Dermatophilosis can be treated by penicillin and streptomycin, tetracycline, chloramphenicol and erythromycin with topical adjuvant therapy by lime sulfur, antibacterial shampoo, chlorhexidine, spraying or dipping with copper or zinc sulphate. Application of acaricides for the control of vectors has also been considered a rational approach (Constable et al. 2017; Hamid and Musa 2019). Unlike treating the infected animal, appropriate control and preventive plans with change in husbandry practice, isolating clinically affected animals, controlling ectoparasites and avoiding direct contact to grooming materials play the major role in maintaining livestock health from this contagious disease (Rebhun and DeLahunta 2019).

Primarily, the disease causes downgrading and rejection of the quality of wool, hides and skin and creates economic consequences due to a decrease in the hides and wool export industry in Ethiopia, in addition to leading to decreased production, reduced fertility and draught performance, treatment, control and preventive costs or losses due to death (Hussen 2020; S. Kumar et al. 2018). Therefore, the current case report describes a case of Dermatophilosis in bull and its treatment outcome.

### 1.1 Study Methods and Description of the Case

**FIGURE 1:**
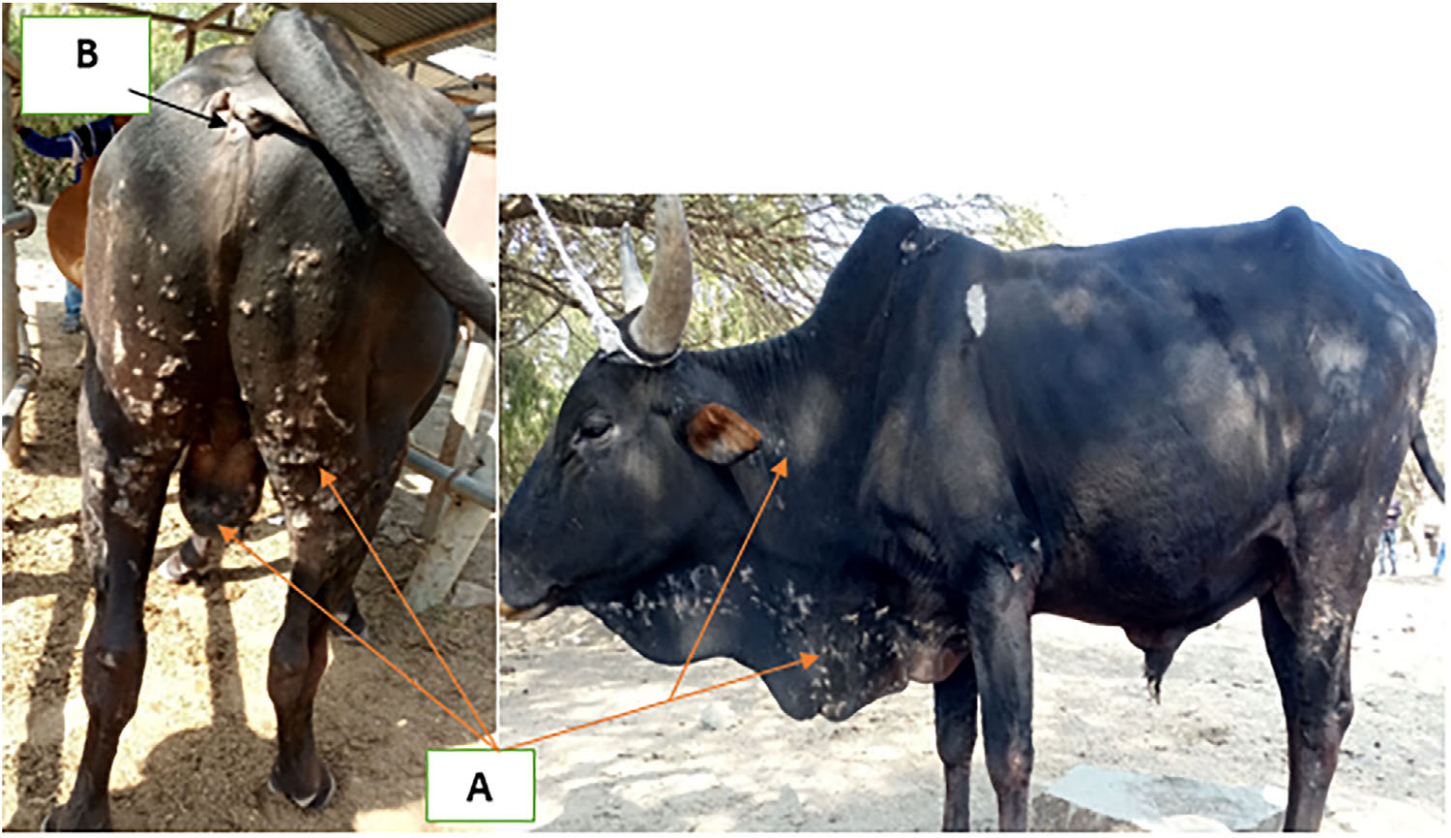
Picture of dermatophilosis suspected bull (A: lesions in different body parts while; B: tick vector at rectal area).

An adult local breed bull with the history of reduced appetite, pruritus, grayish to yellow‐colored scabs and crusts covering different body parts was presented to Debre Markos multipurpose veterinary clinic. Physical examination findings revealed that rectal body temperature (38.9°C), heart or pulse rate (62 beats/min), respiratory rate (18 breaths/min) and capillary refill time were within the normal range with normal color of visible mucus membrane and normal palpable lymph nodes. On clinical examination, the bull showed moderate degree of pruritus. Other signs that include tufted papules initially, matted hair like paintbrush, thick horny crusts projecting from skin that vary in color from grayish to yellow and keratinized material forming wart‐like lesions that were widely distributed mainly on the dorsum (wither), face, neck, distal extremities and scrotum as clearly indicated in Figure 1A were observed. Few ticks (Figure 1B) were noticed on the skin of the bull assumed to be the vector of the disease. Therefore, based on history and clinical sings, the case was tentatively diagnosed as bovine dermatophilosis which is differentially diagnosed from mange and dermatophytosis.

### 1.2 Laboratory Investigation and Results

**FIGURE 2:**
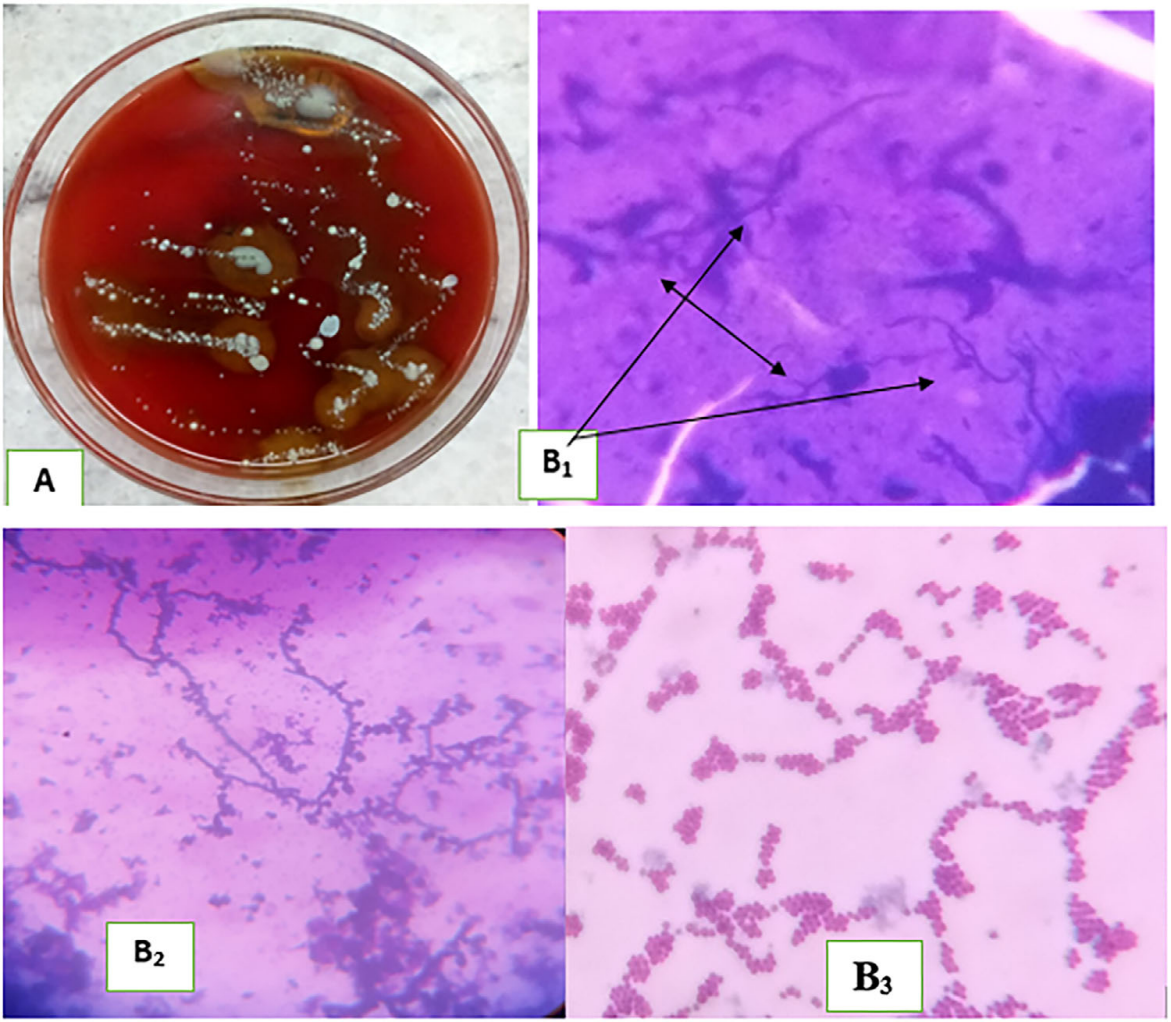
Laboratory result of dermatophylosis suspected bull (A: *D. congolensis* growth on blood agar; B_1_: tram track like appearance on Giemsa stain; B_2_: direct Gram stain result of skin scrap indicating branched hyphae; B_3_: Gram stain result from grown colony on blood agar).

Skin scraping was collected from the affected animal after cleaning the area where the sample was taken and immediately transported to Veterinary Laboratory of Debre Markos University at Debre Markos. Thus, fresh scraps were minced on a sterile pestle and mortal with some drops of sterile distilled water. From this crushed sample, some amount of scrap was taken with a wire loop and inoculated to blood agar base supplemented with 8% sheep blood and incubated at 37°C for 48 h aerobically. It showed growth of *D. congolensis* with small, grayish‐white raised granular, mucoid, hemolytic colonies as indicated in Figure 2A.

Simultaneously, drops of crushed sample were placed on microscope slides and allowed to air dry and stained with Giemsa and Gram's stain. Then, an examination of these stained smears under oil immersion (100X) revealed a result of parallel rows of Gram‐positive branching filament of cocci forms that looked like railroad tracks (tram track like appearance) as clearly indicated in Figure 2B_1_ and B_2_ for Giemsa and Gram staining, respectively. Other Gram staining was also conducted from the grown colony on blood agar for comparison and indicates similar result (Figure 2B_3_).

The biochemical tests were also conducted and indicated catalase and oxidase positive, indole and Voges Proskauer negative and acid production from triple sugar iron. Thus, the above laboratory results indicated the characteristics of* D. congolensis* (etiology of dermatophilosis). Additionally, the skin scrapings were also examined under direct microscopy using 10% Potacium Hydroxide to detect the presence of fungus or mites but did not reveal any positive result. Therefore, based on history, clinical signs and laboratory results, the infection of the bull was confirmed as dermatophilosis.

### 1.3 Case Management and Treatment Outcome

The current case was managed with systemic antimicrobial and topical adjuvant therapy along with change in husbandry practice to keep animals dry. It was treated with penstrep (Interchemie werken “De Adelaar” B.V., Holand) with a recommended dose rate of 1 mL/20 kg body weight for 3 consecutive days intramuscularly and a single dose of ivermectine (Hebei hope harmony pharmaceutical Co., Ltd., China) with the recommended dose of 1 mL/50 kg body weight subcutaneously once a day for infestation of ticks as acaricide. Additionally, iodine tincture was also applied topically for 4 days to hasten recovery along with recommendation of the owner to isolate the animal and keep it in dry place. Following therapy and management of the bull, progression of illness was halted after 4 days therapy, and the animal showed marked improvement, and complete clinical recovery was noticed after 2 months with the gradual disappearance of scabs and crusts as reported by the owner has.

## 2 Discussion

Dermatophilosis is an infectious, highly contagious and economically important skin disease of cattle, sheep and other animals. It is caused by *D. congolensis* which is a normal inhabitant of livestock skin and is found in water, organic material and environment of livestock farms. This disease can be transmitted by direct contact, arthropod vectors and contaminated materials (Bayisa et al. 2012; Walter et al. 2017). Susceptibility of skin by trauma, effect of prolonged or heavy rain fall, malnutrition and concurrent disease, high humidity and temperature, reduced natural barriers of integument of animals and the presence of various ectoparasites are the factors that influence the transmission, prevalence and seasonal incidence of dermatophilosis (Covarrubias 2015; G. Kumar 2020; Ranjith et al. 2018). These authors also stated that the occurrence of the disease varies in different environmental conditions and management systems. It is clearly reported that this disease is highly prevalent in Ethiopia and causes huge economic losses to the wool, skin and hide industry, and it occurs in warm and humid conditions of tropical areas mainly during rainy season or wet weather conditions (Kebede 2019; Sarba and Borina 2017). However, the current case occurred at dry period (mid of January) which can be associated with the presence of tick vectors in the area.

The clinical pictures manifested due to clinical dermatophilosis are matting of the hair or wool, scab and crust formation and generalized formation of massive crust in chronic cases which leads to hair loss and even local loss of the upper skin layers that predisposes to secondary complications. Additionally, the affected animals may present papular crusted lesions that can be initiated by ticks or biting flies which are primarily distributed on different parts of the body of the animal especially on the area of the trunk, head, lower part of the neck, ears, axillae, groin, udder, lower limb and scrotum suggesting the possible involvement of ticks in the establishment of the disease at the same sites (Constable et al. 2017; Sarba and Borina 2017; Siva and Vijaya 2015). This agrees with the current case report in which the presence of tick and clinical lesions of dermatophilosis affecting the bull were noted on different body parts and the signs varied from erythema to thick scab formation which coalesces to form cutaneous keratinized wart‐like lesions.

Many trials have been published on the treatment of dermatophilosis in cattle of different age groups; however, a successful therapy of this disease will give a better outcome when a combination of systemic antibiotics like tetracyclines, penicillins, streptomycin, chloramphenicol, erythromycin, lincomycin‐spectinomycin mixture, ceftiofur, ampicillin and oxytetracycline, and topical application of different preparations were used effectively (Domingues et al. 2018; Hamid and Musa 2019; Ndhlovu and Masika 2016). This is in line with the treatment and management approaches followed for the current case with penstrep which is a combination of penicillin and streptomycin giving a bactericidal effect to the disease‐causing agent and ivermectin for removal of tick and other vectors as acaricide along with topical application of iodine tincture as adjuvant and antiseptic solution. Thus, the bull showed a better clinical improvement and recovery within a short duration of therapy.

## 3 Conclusion and Recommendations

This case report showed that bovine dermatophilosis is an economically important tick‐associated skin disease of cattle characterized by an exudative, pustular crusting dermatitis and formation of scabs, crusts and loss of hair. It is highly prevalent in the study area and other different regions of Ethiopia. This disease can be successfully treated with systemic intramuscular penstrep for the bacteria and subcutaneous ivermectin therapy for the vector (tick) infestation with topical application of iodin tincture in line with recommendations with proper shading practices to control ticks, flies and other vectors infestation. It is strongly recommended that the disease needs early detection and treatment along with following proper husbandry practices to control the losses. Appropriate and timely (seasonal) vaccination is essential for preventive measure in areas where the vaccine is available for use in cattle and sheep. Generally, because dermatophilosis is an economically important skin disease, efforts should be focused on preventing the spread to uninfected animals.

## Author Contributions

**Dessalew Habte**: Conceptualization; investigation; writing—original draft; validation; formal analysis; resources. **Habtamu Addis**: Writing—review and editing; supervision. **Kifle Wondimagegnehu**: Validation; writing—review and editing; resources.

## Conflicts of Interest

The authors declare no conflicts of interest.

### Peer Review

The peer review history for this article is available at https://publons.com/publon/10.1002/vms3.70245.

## Data Availability

The datasets used and/or analyzed during the current study are available from the corresponding author upon reasonable request.
